# Associations of Body Mass Index with Demographics, Lifestyle, Food Intake, and Mental Health among Postpartum Women: A Structural Equation Approach

**DOI:** 10.3390/ijerph17145201

**Published:** 2020-07-18

**Authors:** Hashem Salarzadeh Jenatabadi, Che Wan Jasimah Bt Wan Mohamed Radzi, Nadia Samsudin

**Affiliations:** Department of Science and Technology Studies, Faculty of Science, University of Malaya, Kuala Lumpur 50603, Malaysia; jasimah@um.edu.my (C.W.J.B.W.M.R.); nadiasamz91@gmail.com (N.S.)

**Keywords:** body mass index, postpartum obesity, structural equation modeling

## Abstract

As postpartum obesity is becoming a global public health challenge, there is a need to apply postpartum obesity modeling to determine the indicators of postpartum obesity using an appropriate statistical technique. This research comprised two phases, namely: (i) development of a previously created postpartum obesity modeling; (ii) construction of a statistical comparison model and introduction of a better estimator for the research framework. The research model displayed the associations and interactions between the variables that were analyzed using the Structural Equation Modeling (SEM) method to determine the body mass index (BMI) levels related to postpartum obesity. The most significant correlations obtained were between BMI and other substantial variables in the SEM analysis. The research framework included two categories of data related to postpartum women: living in urban and rural areas in Iran. The SEM output with the Bayesian estimator was 81.1%, with variations in the postpartum women’s BMI, which is related to their demographics, lifestyle, food intake, and mental health. Meanwhile, the variation based on SEM with partial least squares estimator was equal to 70.2%, and SEM with a maximum likelihood estimator was equal to 76.8%. On the other hand, the output of the root mean square error (RMSE), mean absolute error (MSE) and mean absolute percentage error (MPE) for the Bayesian estimator is lower than the maximum likelihood and partial least square estimators. Thus, the predicted values of the SEM with Bayesian estimator are closer to the observed value compared to maximum likelihood and partial least square. In conclusion, the higher values of R-square and lower values of MPE, RMSE, and MSE will produce better goodness of fit for SEM with Bayesian estimators.

## 1. Introduction

High-income countries are often plagued with an obesity pandemic [1]. In that regard, the populations in low- and middle-income countries that are affected by obesity and overweight problems has also risen, especially in urbanized areas [2]. Today, newly emerging public health complications pertaining to women that can affect maternal and child outcomes are pre-pregnancy obesity, excessive gestational weight gain (GWG) [3], and postpartum weight retention (PPWR) [4]. Based on the world population in 2016, an estimated 2.1 billion people were found to be overweight, of which 650 million were obese [5]. It is predicted that half of the population will be affected by obesity and overweight by 2030 [6]. According to the WHO, worldwide obesity has nearly tripled since 1975, with 39% of the adult population being overweight, and 13% suffering from obesity [1].

Obesity can also damage health and reduced lifespan [7,8]. According to previous studies, people with an overweight problem often try to lose weight, but it seems that not many are successful at long-term weight loss maintenance [5]. Obesity could be a risk factor for cardiovascular disease because the accumulation of abdominal fat increases morbidity and mortality from related illnesses [9]. Based on WHO data, more women are reportedly overweight and obese than men [1]. Obesity is a threat to women during the antepartum, peripartum, and postpartum stages [10]. Hence, the world is facing another health issue that is associated with maternal obesity [11].

The pregnancy and postpartum periods are fragile phases for women when they experience weight gain and body structure changes [12]. During pregnancy, approximately 48% of women gain weight excessively [13]. Referring to the guidelines presented by the US Institute of Medicine (IOM), the gestational weight gain (GWG) of women with obesity should be 5.0–9.0 kg [14]. Unfortunately, 20–40% of pregnant women with obesity exceed the GWG recommended by IOM. Obesity in pregnancy might impact the health of both mother and infant in terms of diabetes, hypertensive disorders, preeclampsia, caesarean delivery, high birth weight, preterm delivery, late fetal loss and stillbirth [15,16]. High maternal BMI may lead to postpartum complications, such as continuous weight gain and increased risk of life-long obesity, metabolic syndrome, cardiovascular diseases, and Type 2 diabetes [10,17]. In the early period of postpartum, Butte et al. [18] claimed that the weight of lactating mothers showed a decreasing trend in the first four months postpartum. In order to produce more breast milk, mothers increase their daily diet incessantly, which happened to be one of the reasons postpartum women do not return to their pre-pregnancy weight in less than a year [19].

### 1.1. Previous Studies in Postpartum Obesity Modelling

Few research articles noted and clarified the indicators of obesity and overweight among postpartum women [20,21], and we categorized them into four main groups of indicators related to demographics, food behaviour, health, and lifestyle. The next paragraphs explain how, in previous studies, it was mentioned that those variables were related to their BMI (as a representative of obesity).

The sociodemographic and economic situation are among the main factors that are significant for postpartum women obesity, which is confirmed from previous studies [22,23,24]. Age, education, income, and job experience are the most familiar variables that research scholars are concerned with. In our study, we considered the combination of them in one latent variable as the primary independent variable. Some researchers have found that physical function, sleep quality, average working hours per day, and smoking habits were affecting postpartum obesity. However, there is a lack of studies related to the impact of the average use of screen time on postpartum obesity. These factors represent their lifestyle. In most previous studies regarding postpartum obesity modelling, the effect of those factors on BMI was considered, separately. The factors are related to each other and could be considered as one of the groups of lifestyle variables and are defined as a latent variable. The indicators of lifestyle latent variable (research variable) depend on the participant’s characteristics. The measurement of the variable is defined based on the postpartum lifestyle.

Previous studies like Garmendia et al. [25] reasserted that a factor contributing to women becoming overweight or obese during the postpartum period is an unhealthy lifestyle. Regarding the impact of an unhealthy lifestyle on the postpartum stage, some reviews have highlighted the link between women’s sleep behaviour and physical activity. To emphasize this link, Wen et al. [26] claimed that poor sleep quality would lead women to do less exercise in the postpartum period. This claim was also supported by Waring et al. [27], who indicated that lack of exercise is one of the reasons postpartum women become obese. It is undeniable that some physical activity can prevent excessive gestational and postpartum weight gain [28,29,30]. Variables such as physical activity and sleep behaviour were considered in their research model. In this study, we added screen time, work amount, and smoking habit to the other variables (amount of sleep and physical activity), and they were considered as latent lifestyle variables.

Kay et al. [31] asserted that postpartum obesity is also a result of women’s food consumption behaviour in terms of low consumption of fruits, vegetables, whole grains, and lean protein foods. Parallel to findings on women’s food consumption behaviour, the intake of sugar-sweetened beverages has been linked to postpartum obesity as well. For instance, the findings from a quantitative-based study conducted by Harris et al. [32] showed that many postpartum women like to have sugar-sweetened beverages. These studies were in agreement with Kay et al. [31] regarding the high intake of sugar-sweetened beverages by postpartum women.

Mental disorders also appear to coincide with being overweight and suffering from obesity [33,34]. It is very common for postpartum women to be afflicted with mental disorders as well [35]. Yet studies on the relationship between obesity and mental health seem insufficient so far. Among the existing research on this topic, Teo et al. [36] investigated the correlation between mental health and food intake during the postpartum period, but the variables were not linked with BMI. Hence, limited evidence from previous studies focusing on mental health effects on postpartum obesity could be found.

### 1.2. Aim of the Study and Research Framework

The research framework of this study is the improvement of previous studies on postpartum obesity modelling, and we had involved other research variables obtained from previous literature related to adult and child obesity modelling. Demographics, food intake, and mental health are the most familiar variables in previous studies in postpartum obesity analysis, including the correlation between physical activities with food intake [37], food intake, and sleep quality [38], food intake and health [39]. Therefore, there were some existing studies about the correlation between the lifestyle variables (e.g., sleep quality and physical activity), food behaviour and health. These variables were held to be the most identifiable indicators in the postpartum obesity modelling literature. The elements of lifestyle are one of the leading research variables that are included in the obesity modelling. This was present in some of the previous studies related to children [40] and youth [41]. Those studies showed that the links between the demographics had allowed the BMI to be examined through lifestyle, food intake, and mental health. However, there is a lack of studies that investigate the factors affecting postpartum obesity and that combine the essential variables (lifestyle, food intake, and mental health), which all add to the complexity in acquiring the output of the postpartum women’s BMI.

**Figure 1:**
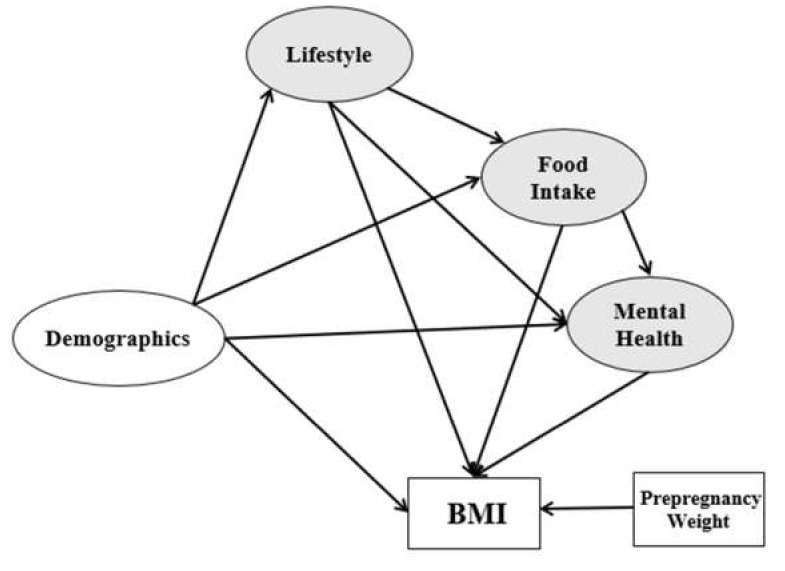
Research framework.

The first contribution of this study was to construct new relationships between the postpartum women’s lifestyle, food behaviour, mental health, and BMI. These relationships were established after the demographics were examined in order to understand the impact of the alleged factors on postpartum obesity. Figure 1 illustrates the conceptual model framework for this research. The framework comprises demographics (initial independent variable), postpartum women’s BMI (main dependent variable), and the remaining variables that function as mediators, known as endogenous and exogenous. The weight pre-pregnancy is added as the control variable, which is consistent with previous literature [42,43].

The following Equations (1)–(4) show statistical regression modeling between the research variables:

(1) $$\mathrm{BMI}={\;\alpha }_{1}\mathrm{Demographics}+\alpha_{2}\mathrm{Lifestyle}+\alpha_{3}\mathrm{Food}\;\mathrm{Intake}+\alpha_{4}\mathrm{Mental}\;\mathrm{Health}\;+\alpha_{5}\mathrm{Prepregnancy}\;\mathrm{Weight}$$

(2) $$\mathrm{Mental}\;\mathrm{Health}={\;\beta }_{1}\mathrm{Demographics}+\beta_{2}\mathrm{Lifestyle}+\beta_{3}\mathrm{Food}\;\mathrm{Intake}$$

(3) $$\mathrm{Food}\;\mathrm{Intake}={\;\gamma }_{1}\mathrm{Demographics}+\gamma_{2}\mathrm{Lifestyle}$$

(4) $$\mathrm{Lifestyle}={\;µ}_{1}\mathrm{Demographics}$$

Obesity modeling with the application of SEM research has been a particular interest of researchers for the last few years. This method enables the estimation of BMI as the dependent variable based on the fundamental relationship between the observed and non-observed variables. Previous studies already introduced a few predictors or estimators for the SEM analysis. In related research on SEM analysis, the maximum likelihood is the most commonly used among all the estimators [44]. Nevertheless, the maximum likelihood estimator is frequently conceded by model misspecification. As an example, poor model fitting results may be produced from the models that are too strict with zero residual correlations and exact zero cross-loadings [45]. A maximum likelihood estimator has been proved by some researchers that demonstrates substantial parameter bias in factor loadings and factor correlation [46,47]. Some researchers already used alternative estimators in modeling to overcome the limitation of maximum likelihood in the SEM analysis due to the small sample size and normal distribution of independent variables. Thus, a few researchers have suggested applying the Bayesian method to overcome the limitations of the maximum likelihood method [44,48]. There seems to be a lack of comparative studies in the literature which examine the patterns that influence postpartum obesity and overweight problem using a multilevel framework that comprises different statistical modeling. Thus, a comparative analysis is currently one of the well-known statistical methods and introduce a better estimator in this dataset would be the second contribution of this study. This approach gives further knowledge about the Bayesian methods of predictive power and also offers opportunities to extend the research.

Several packages within the R language [49,50] provide excellent open source tools for fitting the SEM, including lavaan [51] and OpenMX [52]. Nevertheless, proprietary SEM programs such as Amos [53], LISREL [54], and Mplus [55] enjoy widespread use for a variety of reasons, including ease of use, specialized modeling facilities, and users’ familiarity.

Therefore, the main objective of this study includes two parts. The first part is to explore and develop the previous modeling in postpartum obesity. The second part is to do statistical comparison modeling and introduce a better estimator for the research framework, which is presented in Figure 1.

## 2. Materials and Methods

### 2.1. Sampling

The cross-sectional study design was applied in this research. Accordingly, the data was collected from the research population sample at only one point in time. The researchers had various notions of the sample size. For example, Hair et al. [56] in the theory of structural equation modeling indicated that the sample size is supposed to be related to the number of latent variables in the study, which includes the number of indicators within the latent variables. Table 1 presents Hair et al.’s [56] scheme for choosing the sample size.

For this study, women who were within one year after giving birth were surveyed. We recognized the one-year postpartum period would be a proper time as women usually recover from childbirth, which corresponds to previous studies [57,58]. The respondents were selected randomly from governmental healthcare centres using proportionate stratified random sampling. The survey was conducted with funding from the University of Malaya (project number GPF066B-2018) and approved by the University of Malaya Research Ethics Committee (UM.TNC2/RC/H&E/UMREC 127). The research methods were performed in accordance with the relevant guidelines and regulations. The respondents were provided with an explanation of the research purpose, and informed consent was obtained from all the respondents.

This research considered both urban and rural areas. The main reason the rural and the urban area were chosen was that both areas have different lifestyles and different facilities. Therefore, we preferred to conduct a comparative analysis between the rural and the urban area to understand the significant variables which are effective on BMI. Five main cities of Iran were chosen for the data collection area. Those cities were Tehran, Mashhad, Isfahan, Shiraz, and Tabriz that have the highest population in Iran. For every city, we considered 80 questionnaires for the urban and 30 questionnaires for the rural area. Questionnaires were distributed in some clinics that had agreed to cooperate in this research. From the 550 distributed questionnaires (400 in urban areas and 150 in rural areas), 480 were returned (359 for urban and 121 for rural). The rest refused to participate. From the 480 questionnaires, 28 were eliminated due to missing data (19 urban and nine rural participants). Sixteen (16) bachelor and master students of public health and management were trained for the data collection phase.

The postpartum period considered was one year, and maybe the first one may be varied with respect to the twelfth month. To avoid this issue, we applied an outlier analysis at this point in the study. The Mahalanobis distance is a familiar technique for recognizing outliers in SEM [59]. The Mahalanobis distance output signified that nine observations were outliers, which were consequently eliminated from the data analysis. Therefore, (480 − 28 − 9 = 443) 443 observations were considered as the final sample size for this study.

### 2.2. Research Variable Measurement

We measured every latent variable based on previous studies and adjusted them based on Iran currency and postpartum situation [60].

#### 2.2.1. Demographics (Independent Variable)

Three main measurements were considered for the demographics of the postpartum women: age and work experience. We measured this variable based on previous studies [61,62,63]. Age was classified into five groups, which were below 21, 21 to 25, 26 to 30, 31 to 35, and over 35 years old. Educational background was categorized as less than high school, high school, diploma, Bachelor, and Master or PhD. The respondents’ work experience was categorized according to no job experience, 1 to 3 years, 4 to 6 years, 7 to 10 years, and more than 10 years. The last question in the socio-demographics part was related to household income per month, and the responses were grouped according to less than 2 MT, 2 to 3 MT, 3 to 4 MT, 4 to 5 MT, and over 5 MT (MT: Million Tomans). The results suggest a good fit of specification {[χ^2^] = 55.21, [CFI] = 0.909, [NFI] = 0.959, [TLI] = 0.944, [RFI] = 0.931, [GFI] = 0.908, and [RMSEA] = 0.049}. The value of RMSEA is below 0.050. CFI, NFI, TLI, RFI, and GFI all exceed the recommended threshold level of 0.90 [64]. As a result, demographic measurement model is well-defined.

#### 2.2.2. Lifestyle (First Mediator)

According to Nakayama et al. [65], lifestyle encompasses the average working hours, physical activity, smoking habit, and average sleep hours, which were used in the present study as well. Melzer et al. [66] claimed that smoking habits and physical activity, in particular, are predictors of postpartum obesity. In the current study, one more element was added to the postpartum women’s lifestyle, namely the average hours of screen time (e.g., TV, smartphone, tablet, etc.) to measure the respondents’ social media use [67,68,69]. For measuring average screen time, we asked three questions. These questions: ‘During the past 7 days, how many hours/per day did you watch TV?’, ‘During the past 7 days, how many hours/per day did you spend using a laptop/or computer for watching movies, e-mailing, playing games, etc.?’, and During the past 7 days, how many hours/per day did you spend using a handphone for watching movies, e-mailing, playing games, using (WhatsApp, Facebook, Instagram, etc.), etc.?’ Based on those three questions we calculated the average screen time based on five categories: less than 1 hour, 1 to 2 h, 2 to 3 h, 3 to 4 h, and more than 4 h. The responses for the average working hours per day were in five categories denoted by none, less than 7 h, 7 to 8 h, 8 to 9 h, and more than 9 h. Regarding physical activity, we asked two questions. These questions were: “During the past 7 days, on how many days did you participate in at least 60 min per day of any kind of physical activity that increased your heart rate and made you breath hard, such as fast bicycling, kicking shuttlecock, or aerobic activities, etc.?” and “During the past 7 days, on how many days did you do exercises to strengthen or tone your muscles, such as push-ups, sit-ups, weight lifting, etc.?” [70].

Based on those two questions, we calculated the frequency of physical activity into six categories: none, 1 day, 2 days, 3 days, 4 days, and more than 4 days. Smoking habit was categorized as non-smoker, quit, 1 to 3 cigarettes per day, 4 to 6 cigarettes per day, 7 to 10 cigarettes per day, and more than 10 cigarettes per day. However, in data analysis, the respondents who smoke were grouped as smokers. The average hours of sleep per day were indicated as less than 6 h, 6 to 7 h, 7 to 8 h, 8 to 9 h, and more than 9 h. The results suggest a good fit of specification {[χ^2^] = 48.66, [CFI] = 0.906, [NFI] = 0.933, [TLI] = 0.918, [RFI] = 0.976, [GFI] = 0.938, and [RMSEA] = 0.037}. The value of RMSEA is below 0.050. CFI, NFI, TLI, RFI, and GFI all exceed the recommended threshold level of 0.90 [64]. As a result, lifestyle measurement model is well-defined.

#### 2.2.3. Food Intake (Second Mediator)

There are a few theories for measuring food intake (or food consumption). We combined two theories [71,72] to measure food intake in this study. Information on food intake was collected with 24 h recall from all participants. Nine indicators used for the food intake variables were considered. These included whole grains (grams/day), fruits (grams/day), vegetables (grams/day), sweets (grams/day), chips (grams/day), soft drinks (millilitre/day), fast food (grams/day), processed food (serves/week), Non-processed food (serves/week). For primary measurement analysis, the results did have a good fit of specification {[χ^2^] = 12.66, [CFI] = 0.806, [NFI] = 0.833, [TLI] = 0.918, [RFI] = 0.976, [GFI] = 0.938, and [RMSEA] = 0.089}. Therefore, we applied modification indices for this part of our study. After modification, almost all indices increased and meet the criteria {[χ^2^] = 21.03, [CFI] = 0.921, [NFI] = 0.909, [TLI] = 0.925, [RFI] = 0.982, [GFI] = 0.955, and [RMSEA] = 0.037}.

#### 2.2.4. Mental Health (Third Mediator)

The next latent variable (third mediator) is mental health. Based on Nakayama et al. [65] and Boardman [73] studies, they had proposed these indicators to measure mental health i.e., the serious problems that were faced, stress levels, and happiness in life. These indicators should be determined according to the previous year of the mental state. We chose to use these two theories as this study is measuring mental health generally without medical intervention. This latent variable is been used for the first time to measure the impact of this latent variable on postpartum obesity modeling. Mental health has been used in previous studies but in a different context [41,62]. Yet, there is a lack of studies in postpartum obesity modeling that used mental health as a latent variable.

In this study, the problems they faced were grouped into three categories: no serious problems, 1 or 2 serious problems, and more than two serious problems. Responses to the stress levels were indicated by the normal, medium, and high levels of stress. Happiness in life was categorized as not happy, average, and happy. The results suggest a good fit of specification {[χ^2^] = 38.98, [CFI] = 0.939, [NFI] = 0.943, [TLI] = 0.918, [RFI] = 0.977, [GFI] = 0.952, and [RMSEA] = 0.046}. The value of RMSEA is below 0.050. CFI, NFI, TLI, RFI, and GFI all exceed the recommended threshold level of 0.90 [64]). As a result, mental health measurement model is well-defined.

#### 2.2.5. BMI (Dependent Variable)

To measure the BMI of an individual, the weight and height indicators need to be calculated as follows [74]:

(5) $$\mathrm{BMI}=\frac{\left(\mathrm{Weight}\;\mathrm{in}\;\mathrm{kilograms}\right)}{{\left(\mathrm{Height}\;\mathrm{in}\;\mathrm{meters}\right)}^{2}}$$

For postpartum women, the BMI was determined according to standard measurement [75]. In this study on postpartum obesity, Table 2 serves as a reference for BMI measurement.

Postpartum women need to know their weight and height to enable for their BMI to be determined. Thus, with knowledge of the postpartum women’s BMI, it was possible to clearly show the interconnections between latent and observable variables in this research.

### 2.3. Statistical Method

To aim is to analyze the relationship between numerous factors and BMI in postpartum obesity analysis, descriptive statistics, regression, MANOVA, and ANOVA, which are the most well-known applications used from a mathematical and statistical modeling point of view.

SEM is beneficial in this research for providing a better understanding of the concept of latent variables with their functions within the model. In other words, the ability to use latent variables. According to Bollen [76], “latent variables provide a degree of abstraction that permits us to describe relations among a class of events or variables that share something in common.” A precise typical of SEM is the usage of ‘latent variables,’ which are not applied in any other statistical modeling. Latent variables refer to constructs that are not directly observable. For instance, in this study, lifestyle is regarded as a latent variable, which is defined as a combination (average or sum) of observed variables including physical activity, smoking habit, and average sleep hours as well as screen time.

### 2.4. Data Analysis Software

Analyses were performed using SPSS (version 25, SPSS Inc., Chicago, IL, USA) in the first part of data analysis, i.e., descriptive statistics analysis. While the AMOS software (IBM, Armonk, NY, USA) was applied in the second part of data analysis for the structural equation modeling analysis.

## 3. Results

### 3.1. Descriptive Analysis

Table 3 shows the distribution of the respondents based on location. The majority of urban respondents are diploma holders, whereas most rural respondents only have a high school education. For the highest monthly income, 38.2% of urban respondents receive 4–5 MT per month, and 60.2% of the rural respondents receive 2–3 MT per month. The smallest number of rural respondents have no job experience, and none has ever worked for more than 10 years. Many urban and rural respondents have worked for 4–6 years and 1–3 years, respectively. Besides, 25.7% of the study sample from the urban regions participate in physical activity 3 times a week, but 38.9% of the rural participants report that they take part in physical activity only once a week. The highest screen time per day for the urban sample is 44.5%, and for the rural sample, their screen time is 58.3%, which translates into 2–3 and 1–2 h a day, respectively. As for sleep, most participants from the urban and rural areas sleep respectively around 7–8 and less than 6 h per day. Yet the majority (63.3%) of the urban postpartum women work 8–9 h a day, and 54.6% of the rural postpartum women work more than 9 h a day. It was also found that 51.6% of the urban participants are smokers, and 78.7% of the rural participants are non-smokers. Based on Table 3, 7.8% of the respondents (26/335) from the urban study sample are underweight, 22.7% (76/335) are in the normal range, 55.2% (185/335) are overweight, and 14.3% (48/335) suffer from obesity. In rural areas, 11.1% (12/108) of the respondents are underweight, 47.2% (51/108) are in the normal range, 30.6% (33/108) are overweight, and 11.1% (12/108) have obesity.

The data were collected in the urban and rural areas in Iran via questionnaires that were distributed to a total of 335 urban participants and 108 rural participants, with no missing data. Thus, the data for this study were tabulated in the descriptive statistical analysis.

The distribution of different types of food intake is presented in Table 4. Whole grains (bread, rice, pasta, noodles, breakfast cereals) among the urban sample are equal to 246.22 ± 83.69 g per day. As for the rural sample it is equal to 241.34 ± 89.56 g per day.

### 3.2. SEM Analysis

#### 3.2.1. Validity and Reliability

The validity and reliability of a questionnaire are dependent on some conditions of SEM analysis based on Fornell et al. [77]:

(a) Validity:
  - The Cronbach’s alpha value must be equal to or higher than 0.7 for every latent variable in the study.
(b) Reliability:
  - The factor loading of the indicator on each latent variable should be higher than 0.70.
  - The average variance extracted (AVE) for all the latent variables should be equal to or higher than 0.50.

Table 5 tabulates the factor loading, AVE, and Cronbach’s alpha of the demographics, lifestyle, food intake, and mental health variables. The table denotes that age, work, and vegetables obtained factor loading values of below 0.7. These indicators should therefore be excluded from the SEM analysis. However, we are supposed to apply moderation analysis to the comparison analysis between the urban and rural variables. Therefore, for better accuracy, we keep all of the indicators for the next level of SEM analysis. The Average Variance Extracted (AVE) analysis output is illustrated in Table 5, which demonstrates that all the latent variables’ AVE values are greater than 0.5. Since the reliability conditions are fulfilled, the study is accepted. Moreover, Table 5 displays the Cronbach’s alpha output with four latent variables whose indices are higher than 0.7. According to the validity conditions applicable to the analysis, the research model is accepted.

#### 3.2.2. Normality Testing

We considered Anderson-Darling and Shapiro-Wilk statistics for the normality test. All of their *p*-values are less than 0.05, and the normality of the variables is rejected Therefore, SEM with maximum likelihood estimator is not suitable for the modelling of our data (see Table 6).

#### 3.2.3. Model Fitting

**Figure 2:**
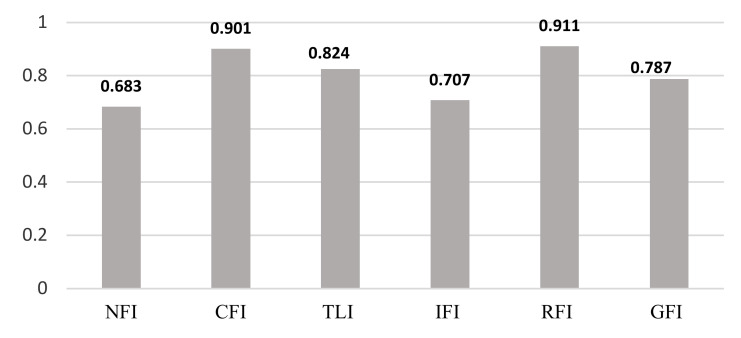
Model fit analysis.

Figure 2 illustrates the model fitting output according to the raw data of SEM. The acceptable model fit values are above 0.9, and the normed fit index (NFI), comparative fit index (CFI), Tucker Lewis index (TLI), incremental fit index (IFI), relative fit index (RTI) and goodness of fit index (GFI) values are all evidently acceptable.

**Figure 3:**
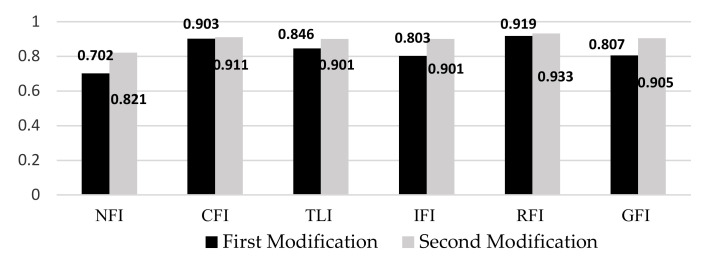
Model fit analysis after modification indices analysis.

Based on Figure 2, from the four indices, four of them (NFI, TLI, IFI, and GFI) obtain less than 0.9. Therefore, model fitting is not accepted for the raw data. In order to solve this issue, modification indices are used. Modification indices are applied to create alternative models to improve fitting. But, they must be supplemented with sufficient reasons based on theoretical justification [78]. In addition, Silvia et al. [79] suggested that it should be minimized to avoid over-fitting in the modeling analysis. We applied the times’ modification indices. Figure 3 shows the model fitting analysis after we applied the first and the second modification indices in our modeling process.

### 3.3. Comparison Analysis among Different SEM Estimators

This section presents a comparative analysis of the SEM with maximum likelihood estimator, partial least square in estimating the BMI in the obesity framework. Four indices were used to compare the two prediction methods: coefficient of determination (R^2^), root mean square error (RMSE), mean absolute error (MSE) and mean absolute percentage error (MPE). These are the most common statistical indices for modeling evaluation, and they are clarified by the following equations:

(a) Coefficient of Determination (R^2^)

(6) $$R^{2}=\frac{{\left[\sum_{i=1}^{n}\left(y_{i}^{\prime }-{\bar{y}}_{i}^{\prime }\right).\left(y_{i}-{\bar{y}}_{i}\right)\right]}^{2}}{\sum_{i=1}^{n}\left(y_{i}^{\prime }-{\bar{y}}_{i}^{\prime }\right).\sum_{i=1}^{n}\left(y_{i}-{\bar{y}}_{i}\right)}$$

(b) Root mean square error (RMSE)

(7) $$\mathrm{RMSE}=\sqrt[{2}]{\frac{\sum_{i=1}^{n}{\left(y_{i}^{\prime }-y_{i}\right)}^{2}}{n}}$$

(c) Mean absolute error (MSE)

(8) $$\mathrm{MSE}=\frac{\sum_{i=1}^{n}\left|y_{i}^{\prime }-y_{i}\right|}{n}$$

d) Mean absolute percentage error (MPE)

(9) $$\mathrm{MAPE}=\frac{1}{n}\;\sum_{i=1}^{n}\left|\frac{y_{i}^{\prime }-y_{i}}{y_{i}}\right|$$

In the formula above, $y_{i}$ is the i^th^ actual value of the dependent variable and $y_{i}^{\prime }$ is the i^th^ predicted value. Table 7 presents the values of the four performance indices, including R^2^, RMSE, MSE, and MPE for maximum likelihood, partial least square, and the Bayesian estimators. The R^2^ value for SEM with the Bayesian estimator is more significant than that for partial least square and maximum likelihood estimators. Moreover, the values of RMSE, MSE, and MPE for the Bayesian estimator are lower than those for the maximum likelihood and partial least square estimators. Therefore, the performance indices for the SEM with the Bayesian estimator indicate a superior estimation compared to SEM with a partial least square estimator or SEM with maximum likelihood estimator.

### 3.4. Structural Model

**Figure 4:**
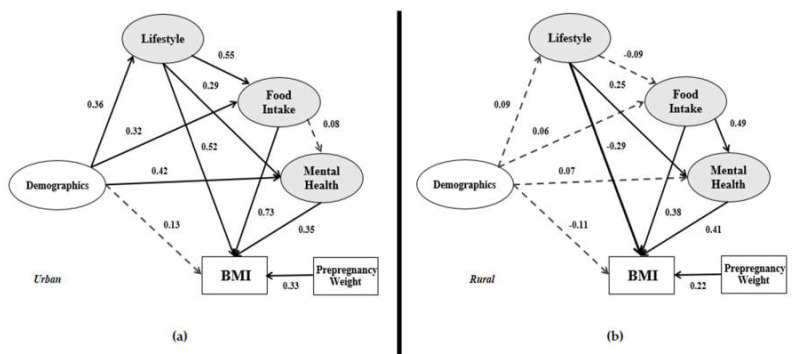
(**a**) Urban structural model; (**b**) Rural structural model.

With the SEM technique, the structural model serves to recognize the hypothesized relationships between the variables that show the links to the presumed model’s conception. Figure 4 presents urban and rural structural models.

Figure 4 shows the structural models i.e., (a) urban structural model and (b) rural structural model. From the ten relationships in the urban model, demographic details has a significant impact on lifestyle (β = 0.36), food intake (β = 0.32) and mental health (β = 0.42). However, the impact of demographics on these three variables in the rural model is not significant. It is also observed in both models where demographics did not have a significant impact on the dependent variable. It means that demographics do not have a significant direct impact on BMI. In the urban model, demographics have a significant indirect impact on BMI through lifestyle, food intake, and mental health. The lifestyle variable in the urban model has a positive significant relationship with food intake (β = 0.55), mental health (β = 0.29) and positive significant impact on BMI (β = 0.52). It has an indirect impact on BMI through food intake and mental health. In the rural model, lifestyle has a significant impact only on mental health, and there is no indirect impact on BMI. According to the urban and rural models in Figure 4, the impact of food intake on postpartum women’s BMI is significant (β = 0.73 and β = 0.38, respectively). Nonetheless, food intake has a more significant and positive impact on mental health in the rural model compared to the urban model. The indirect impact of food intake on BMI is confirmed in the rural model. As for the last variable, mental health is said to have the same significant impact on BMI in both models.

### 3.5. Mediation Analysis

**Figure 5:**
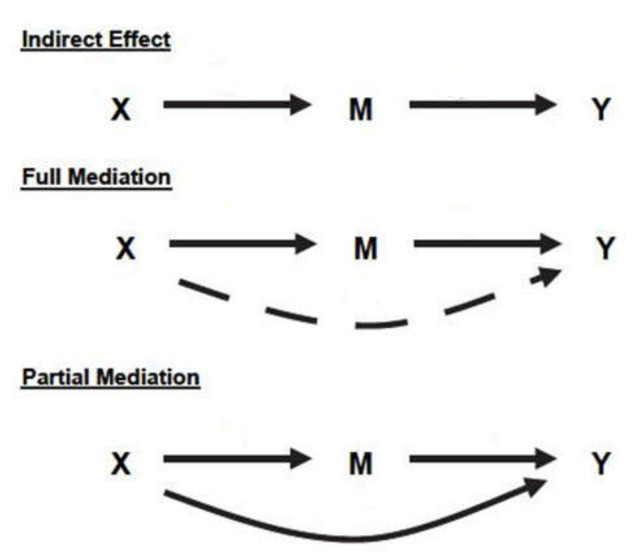
Comparing indirect with full and partial mediation effects.

When a mediator exists between independent and dependent variables, the relations may be characterized as an indirect effect, partial mediation, or full mediation, which are defined as follows (see Figure 5):

- Indirect effect: There is no relation between X and Y, and both X → M, M → Y have a significant relationship.
- Partial mediation: X → M, M → Y, and X →Y have a significant relationship.
- Full mediation: X → M and M → Y have a significant relationship, and X →Y has no significant relationship.

Based on Figure 4 and Table 8, from both models, the demographics have no significant impact on BMI. Therefore, none of the lifestyle, food intake, and mental health are mediators between the demographics (independent variable) and BMI (dependent variable). However, the indirect effect of Demographics → Lifestyle → BMI, Demographics → Food intake → BMI, and Demographics → Mental health → BMI have significant effects on the urban model. Therefore, the urban model demographics has an indirect effect on BMI through lifestyle, food intake, and mental health.

**Figure 6:**
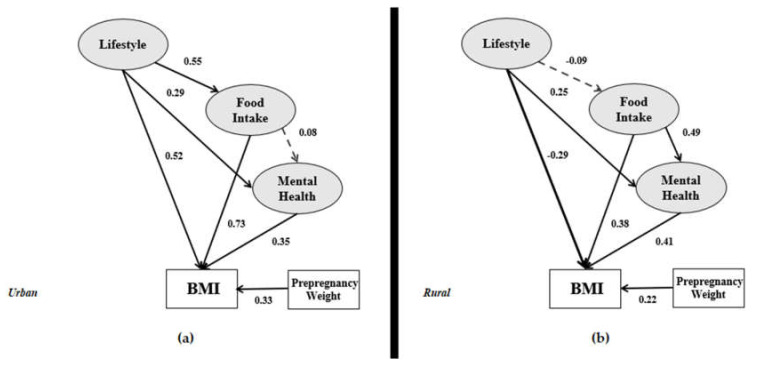
Lifestyle correlation in the (**a**) urban and (**b**) rural research frameworks.

Figure 6 and Table 9 presents the relationship of lifestyle in the research models. Both models show that lifestyle has a significant effect on BMI directly and indirectly through mental health. Therefore, mental health in both models is a mediator between lifestyle and BMI. Only in the urban model, food intake is a mediator between lifestyle and BMI. Therefore, in the urban model, food intake and mental health are mediators between lifestyle and BMI.

**Figure 7:**
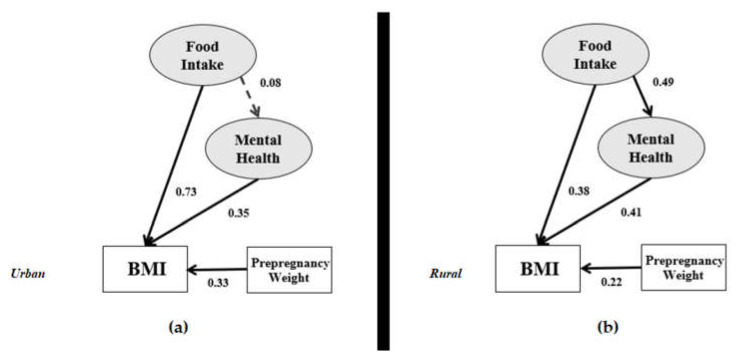
Impact of food intake in the (**a**) urban and (**b**) rural research frameworks.

Figure 7 provides a clear view of the food intake variable associated with BMI in the urban and rural models. Food intake does not exhibit the probability of mental health in the urban model, but it correlates with mental health in the rural model (0.49 × 0.41 = 0.200 and significant). Therefore, specifically for the rural model, mental health is a mediator between food intake and BMI.

### 3.6. Moderation Analysis

Chin Test is based on the previous study [80] that suggested the moderation analysis via the *t*-test with the following formula:

(10) $$t=\frac{{\mathrm{Regression}\;\mathrm{Coeficient}}_{\mathrm{group}1}-{\;\mathrm{Regression}\;\mathrm{Coeficient}}_{\mathrm{group}2}}{\left[\sqrt{\frac{{\left(m-1\right)}^{2}}{\left(m+n-2\right)}*S.E._{\mathrm{group}1}^{2}+\frac{{\left(n-1\right)}^{2}}{\left(m+n-2\right)}*S.E._{\mathrm{group}2}^{2}}\right]*\left[\sqrt{\frac{1}{m}+\frac{1}{n}}\right]}\;$$

Table 10 presents the t-values calculated based on the Chin test, with a significance level of 5%, above the critical t-value with n + m − 2 degrees of freedom for nearly all the correlations (t-value > 1.98).

*Note 2*. Multivariate analysis of variance (MANOVA) is simply an ANOVA with several dependent variables. Based on Figure 1 we have one dependent variable. For the comparison study between SEM and MANOVA, we considered four models based on BMI level. Model 1 is the representative of modeling based on the underweight group, Model 2 is the representative modeling for the normal group, Model 3 is the representative modeling for the overweight group, and Model 4 is the representative for the obese group.

Table 11 shows the outputs of the R-Square and RMSE of SEM and MANOVA. Based on Table 11, it is illustrated that the R-Squares of SEM for the four models are higher than MANOVA while the RMSE values of the SEM technique are less than MANOVA.

## 4. Discussion

This paper aimed to introduce a new framework and examine the relationships between postpartum obesity factors and BMI by applying different SEM estimators. Postpartum women’s BMI was the dependent variable and demographics was the main independent variable. Between the demographics and BMI, three latent variables were identified based on previous studies. Hence, an enhanced study model was designed accordingly from previous theories and frameworks of postpartum obesity modelling. The research framework is shown in Figure 1. Two types of data are allotted in the overall dataset, which involves the BMI for urban and rural areas, as per Figure 6. The interrelationships among the demographics, lifestyle, and mental health in the urban model are very strong, as confirmed in a study conducted by Widen et al. [81]. However, these interrelationships in the rural model are weakened by the non-significant impact of demographics on the lifestyle variable.

The impact of demographics on BMI has previously also been confirmed by Hill et al. [82], Ghee [83], and Waring et al. [27]. These preceding studies showed that the indicators of demographics, such as income and working experience, affect the BMI category output, namely being underweight, normal, overweight, and obese. However, the present data analysis in Figure 6 demonstrates that the involvement of demographics (i.e., age, educational background, work experience, and income) on BMI is not significant in both urban and rural models (β = 0.13 and β = −0.11, respectively).

Nevertheless, the lifestyle variable in the urban model has been associated with food intake, mental health, and BMI variables. Lifestyle is, therefore, correlated with the dependent variables of this research model. Concerning the factor loading analysis in Table 5, the lifestyle indicator shows that screen time and sleep have the highest factor loadings in the urban areas. In the rural model, the lifestyle variable has a significant impact only on mental health and BMI. Therefore, lifestyle is also directly associated with the BMI variable (β = −0.29). The factor analysis in Table 5 highlights that the sleep indicator followed by the work indicator have the highest factor loadings among the lifestyle latent variables. The sleep indicator seems to be very important in the lifestyle factor analysis. A previous study also investigated and found the sleep indicator’s effect on BMI [26]. Women typically do less physical activity during pregnancy [20,84]. Still, evidence [4] suggests that light to moderate-intensity physical activity, including aerobic and resistance exercise should be encouraged for 30–60 min, 3–5 times per week without adverse effects in healthy pregnant women.

Many postpartum women often fail to consume a balanced diet, influenced by cultural, psychological, and economic factors. Due to hindrances in healthy eating, especially during the postpartum period, the risk of becoming overweight or obese increases. Previous studies have shown that excessive weight gain in the year following childbirth is caused by maternal obesity. Besides, pregnancy with obesity also intensifies food insecurity and lowers the quality of food intake [20]. The path coefficient between food intake and BMI in both models denotes a significant relationship, which has been reported in prior studies [20,85]. Based on the factor analysis (Table 5) for urban areas, the soft drinks indicator has the highest value among the indicators. A prior study also claims that sugar-sweetened beverages (e.g., soft drinks) impact the BMI of postpartum women [31]. Meanwhile, the rural factor analysis for food intake signifies that fruit intake has the highest value. From the highest factor loadings in both models, it is evident that urban postpartum women consume more unhealthy food, and rural postpartum women consume more healthy food.

Referred to the value of R^2^ in Table 7, we can interpret that SEM with Bayesian estimator of 81.1% variation in BMI of postpartum women is related to demographics, lifestyle, food intake, and mental health. However, this variation, based on SEM with partial least square estimator and SEM with maximum likelihood estimator, is equal to 70.2% and 76.8%, respectively. Based on the output of RMSE, MSE, and MPE, the residuals value of the SEM with Bayesian estimator are less than the other two estimators, which means that the predicted values of SEM with Bayesian estimator are closer to the observed value than the maximum likelihood and partial least square. As a result, with higher values of R^2^ and lower values of MPE, RMSE, and MSE, SEM with the Bayesian estimators have better goodness of fit for the observations.

Figure 4 illustrates a different input-output model structure for urban and rural area data. Nevertheless, some limitations of the current study are as follows:

(1) In previous studies, the parity of mothers [86] was found to be one of the factors that lead to postpartum obesity and should be included in a model. Therefore, the suggestion to study this factor in future research on account of its significance is proposed.
(2) There are other indicators that logically affect postpartum obesity, such as the number of calories consumed per day and the participants’ knowledge of calorie intake. However, this article only addressed food intake but not calories.
(3) In this study, the weight, height, and some parts of the food intake of the respondents were self-reported. Based on previous studies had used the same method, we believe the data provided are valid and considerable [87,88,89]. Other than that, information was collected within 24 h recall from all participants. To reduce type II error, our data collection team coached participants on how to measure our request variables with high accuracy.
(4) Food Frequency Questionnaire (FFQ), International Physical Activity Questionnaire (IPAQ), Groningen Sleep Quality Scale (GSQS) are the most familiar measurement tools to measure food intake, physical activity, and sleep quality. We had used these questionnaires in our pilot study. However, we have less than 10% of participants responded to our request, therefore, we chose to use other theories for measuring food intake, physical activity, and sleep quality for this study.
(5) There might be some additional treatments that may have been received by the respondents. Some respondents may also receive treatments from psychiatrists, psychologists, personal trainers, and medicines that may have contributed to the results obtained. Their treatment history might be affecting the BMI among the respondents, so it would be beneficial for future research to correlate these indicators with weight management after pregnancy.
(6) Activity in front of the screen was not included in this study’s measurement. This is because a person spending hours in front of the screen reading books, or playing games is not the same as those spending hours on social media. Previous research [90] proves that smartphone addiction influences the evaluation of mental health, i.e., depression. We would suggest that researchers consider this criterion in future research.
(7) Our study is limited in terms of being a cross-sectional survey. As such, it has the deficiency of measuring a single moment, which is not convenient when analyzing variables such as mental health. This is because these variables can be affected by a chronic state of obesity and being in a poor family network. To provide more confidence in the accuracy of the SEM output, we suggest pairing the proposed model with longitudinal data.

Consequently, three recommendations are made for practice or policymaking to improve postpartum women’s weight and prevent postpartum obesity. First, this study sought to practically attract the attention of health professionals to provide postpartum women affected by obesity with suitable healthcare follow-up. By understanding what postpartum women with overweight or obese feel and think, clinical intervention could help them live a healthier lifestyle. These women would also be more determined to change if the health teams provide a listening, welcoming, and counselling environment. Second, healthcare professionals should disseminate the latest updates on obesity risks more effectively and frequently via all possible social media platforms. In this era of information and communications technology (ICT), social media makes the perfect platform to spread knowledge to people, raising a massive awareness about obesity. Third, policymakers need to strengthen their strategies for obesity prevention by making them more user-friendly to resonate with women’s daily routines, especially mothers. Adapting more comfortable and convenient policies may help people change and become healthier.

## 5. Conclusions

Obesity not only burdens healthcare systems globally, but it can also potentially impair national economies. Young and adult women appear to be at increased risk of substantial weight gain. Pregnancy has frequently been cited as a contributor to overweight and obesity problems in women, creating a worldwide public health challenge. Women undergo unpleasant experiences throughout their reproduction cycle, including pregnancy, lactation, and childcare. These may have significant impacts on women’s health in terms of parity, obesity, and non-communicable diseases. This article reviewed literature that examines the development of overweight and obesity after pregnancy. It is generally agreed that there is a great probability that high BMI pregnancies carry the risk of experiencing postpartum overweight or obesity. The new framework for postpartum obesity modelling presented in the current study is an improvement from previous studies on food intake, lifestyle, and mental health with a focus on postpartum women’s weight.

In this research, we examined three types of SEM estimators. We became aware that SEM also has some advantages in comparing the regression modelling, MANOVA, and Spearman correlation. However, SEM has some limitations in terms of the normality of the research variable, sample size, and some of the variables are not able to be included in the research model.

## Figures and Tables

**Table 1:** Minimum sample size in SEM analysis.

Sample Size Required	Criteria from the Research Model
100 participants	Consists of five or less latent variables, each of which has at least three indicators
150 participants	Consists of seven or less latent variables, each of which has at least three indicators
300 participants	Consists of seven or less latent variables, each of which has less than three indicators
500 participants	Consists of more than seven latent variables, each of which has less than three indicators

**Table 2:** BMI categories.

BMI (kg/m^2^)	Category
<18.5	Underweight
18.5–24.9	Normal
25.0–29.9	Overweight
≥30.0	Obese

**Table 3:** Demographics, lifestyle, mental health, and BMI of respondents by area.

	Number			Percentage		
	Total	Urban	Rural	Total	Urban	Rural
Age (n, %)						
21 to 25 years old	30	21	9	6.8%	6.3%	8.3%
26 to 30 years old	99	74	25	22.3%	22.1%	23.1%
31 to 35 years old	173	132	41	39.1%	39.4	38.0%
Over 35 years old	141	108	33	32.2%	32.2%	30.6%
Education (n, %)						
Less than high school	26	5	21	5.9%	1.5%	19.4%
High school	119	75	44	26.9%	22.4%	40.7%
Diploma	151	124	27	34.1%	37.0%	25.0%
Bachelor	101	89	12	22.8%	26.6%	11.1%
Master or PhD	46	42	4	10.4%	12.5%	3.7%
Income (n, %)						
<2 MT ($200)	80	43	37	18.1%	12.8%	34.3%
2–3 MT ($200–300)	111	46	65	25.1%	13.7%	60.2%
3–4 MT ($300–400)	81	76	5	18.3%	22.7%	4.6%
4–5 MT ($400–500)	129	128	1	29.1%	38.2%	0.9%
>5 MT ($500)	42	42	0	9.5%	12.5%	0.0%
Job Experience (n, %)						
No job experience	58	26	32	13.1%	7.8%	29.6%
1–3 years	111	62	49	25.1%	18.5%	45.4%
4–6 years	191	166	25	43.1%	49.6%	23.1%
7–10 years	53	51	2	12.0%	15.2%	1.9%
>10 years	30	30	0	6.8%	9.0%	0.0%
Physical Activity (n, %)						
None	105	71	34	23.7%	21.2%	31.5%
1 day/week	105	63	42	23.7%	18.8%	38.9%
2 days/week	99	67	32	22.3%	20.0%	29.6%
3 days/week	86	86	0	19.4%	25.7%	0.0%
4 days/week	36	36	0	8.1%	10.7%	0.0%
>4 days/week	12	12	0	2.7%	3.6%	0.0%
Screen Time (n, %)						
<1 hour/day	40	16	24	9.0%	4.8%	22.2%
1–2 h/day	104	41	63	23.5%	12.2%	58.3%
2–3 h/day	170	149	21	38.4%	44.5%	19.4%
3–4 h/day	88	88	0	19.9%	26.3%	0.0%
>4 h/day	41	41	0	9.3%	12.2%	0.0%
Sleep (n, %)						
<6 h/day	64	3	61	14.4%	0.9%	56.5%
6–7 h/day	75	36	39	16.9%	10.7%	36.1%
7–8 h/day	200	192	8	45.1%	57.3%	7.4%
8–9 h/day	49	49	0	11.1%	14.6%	0.0%
>9 h/day	55	55	0	12.4%	16.4%	0.0%
Work (n, %)						
None	35	33	2	7.9%	9.9%	1.9%
<7 h/day	25	19	6	5.6%	5.7%	5.6%
7–8 h/day	48	36	12	10.8%	10.7%	11.1%
8–9 h/day	241	212	29	54.4%	63.3%	26.9%
>9 h/day	94	35	59	21.2%	10.4%	54.6%
Smoking habit (n, %)						
Non-smoker	193	108	85	43.6%	32.2%	78.7%
Quit	64	54	10	14.4%	16.1%	9.3%
Smoker	186	173	13	42.0%	51.6%	12.0%
Serious problem (n, %)			
0	125	92	33	28.2%	27.5%	30.6%
1–2	134	105	29	30.2%	31.3%	26.9%
>2	184	138	46	41.5%	41.2%	42.6%
Stress (n, %)			
Normal	109	88	21	24.6%	26.3%	19.4%
Medium	118	86	32	26.6%	25.7%	29.6%
High	216	161	55	48.8%	48.1%	50.9%
Happiness (n, %)			
Happy	214	162	52	48.3%	48.4%	48.1%
Average	173	133	40	39.1%	39.7%	37.0%
Not happy	56	40	16	12.6%	11.9%	14.8%
BMI (kg/m^2^)						
Underweight (n, %)	38	26	12	8.6%	7.8%	11.1%
Normal (n, %)	127	76	51	28.7%	22.7%	47.2%
Overweight (n, %)	218	185	33	49.2%	55.2%	30.6%
Obese (n, %)	60	48	12	13.5%	14.3%	11.1%

**Table 4:** Descriptive statistics of food intake (Mean ± Std).

Whole Grains (g/day)	246.22 ± 83.69	241.34 ± 89.56
Fruits (g/day)	392.11 ± 120.49	402.20 ± 118.43
Vegetables (g/day)	323.94 ± 99.38	328.88 ± 93.80
Sweets (g/day)	130.79 ± 29.25	126.56 ± 29.03
Chips (g/day)	90.96 ± 34.98	86.82 ± 35.36
Soft Drinks (ml/day)	385.53 ± 129.80	362.35 ± 130.69
Fast Food (g/day)	213.99 ± 96.81	196.37 ± 99.33
Processed Food (serves/week)	4.50 ± 2.17	3.81 ± 1.93
Non-Processed Food (serves/week)	2.52 ± 1.74	2.31 ± 1.62

**Table 5:** Validity and reliability output.

Parameter Description	Urban	Rural	Total
**Demographics (AVE = 0.51; Cronbach’s Alpha = 0.72)**			
Age	0.43	0.56	0.51
Education	0.81	0.62	0.76
Income	0.76	0.73	0.73
Job Experience	0.79	0.71	0.75
**Lifestyle (AVE = 0.53; Cronbach’s Alpha =0.73)**			
Physical Activity	0.73	0.53	0.77
Screen Time	0.86	0.49	0.81
Sleep	0.74	0.86	0.73
Work	0.62	0.79	0.59
Smoking habit	0.71	0.52	0.79
**Food Intake (AVE = 0.69; Cronbach’s Alpha =0.77)**			
Whole Grains (g/day)	0.56	0.73	0.71
Fruits (g/day)	0.72	0.83	0.81
Vegetables (g/day)	0.67	0.81	0.62
Sweets (g/day)	0.85	0.72	0.77
Chips (g/day)	0.88	0.61	0.79
Soft Drinks (ml/day)	0.92	0.59	0.88
Fast Food (g/day)	0.81	0.54	0.85
Processed Food (servs/week)	0.81	0.82	0.88
Non-Processed Food (servs/week)	0.78	0.71	0.76
**Mental Health (AVE = 0.55; Cronbach’s Alpha =0.71)**			
Stress Level	0.86	0.71	0.88
Happiness	0.84	0.72	0.89
Problems	0.88	0.66	0.86

**Table 6:** Normality Test.

Variables	Anderson-Darling Test		Shapiro-Wilk Test	
	Statistic	*p*-value	Statistic	*p*-value
Age	26.053	<0.001	0.850	<0.001
Education	16.503	<0.001	0.911	<0.001
Income	17.301	<0.001	0.895	<0.001
Job Experience	15.315	<0.001	0.904	<0.001
Physical Activity	13.980	<0.001	0.907	<0.001
Screen Time	16.011	<0.001	0.913	<0.001
Sleep	20.261	<0.001	0.889	<0.001
Work	40.276	<0.001	0.783	<0.001
Smoking habit	54.045	<0.001	0.719	<0.001
Whole Grains (g/day)	6.964	<0.001	0.943	<0.001
Fruits (g/day)	6.413	<0.001	0.946	<0.001
Vegetables (g/day)	4.374	<0.001	0.959	<0.009
Sweets (g/day)	5.220	<0.001	0.953	<0.005
Chips (g/day)	5.822	<0.001	0.950	<0.001
Soft Drinks (mL/day)	5.915	<0.001	0.951	<0.001
Fast Food (g/day)	5.477	<0.001	0.949	<0.001
Processed Food (servings/week)	9.440	<0.001	0.933	<0.001
Non-Processed Food (servings/week)	12.573	<0.001	0.904	<0.001
Stress Level	45.522	<0.001	0.754	<0.001
Happiness	45.515	<0.001	0.761	<0.001
Problems	39.435	< 0.001	0.779	< 0.001

**Table 7:** Comparison analysis among different SEM estimators.

	Statistical Indices			
	R^2^	RMSE	MSE	MPE
SEM-Partial Least Square	0.736	4.166	0.121	0.093
SEM-Maximum Likelihood	0.781	3.151	0.109	0.059
SEM-Bayesian	0.823	2.118	0.089	0.039

**Table 8:** Mediation analysis of lifestyle, food intake, and mental health between demographics and BMI.

	Direct	Indirect	Total	Result
**Urban**				
Mediation of lifestyle	0.13	0.36 × 0.52 = 0.182 *	0.312 *	Indirect effect
Mediation of food intake	0.13	0.32 × 0.73 = 0.233 *	0.363 *	Indirect effect
Mediation of mental health	0.13	0.42 × 0.35 = 0.147 *	0.277 *	Indirect effect
**Rural**				
Mediation of lifestyle	−0.11	0.09 × −0.29 = −0.026	−0.136	-
Mediation of food intake	−0.11	0.06 × 0.38 = 0.022	−0.132	-
Mediation of mental health	−0.11	0.07 × 0.41 = 0.028	−0.138	-

In this Table * is representative of significant relationship.

**Table 9:** Mediation analysis of food intake and mental health between lifestyle and BMI.

	Direct	Indirect	Total	Result
**Urban**				
Mediation of food intake	0.52 *	0.55 × 0.73 = 0.401 *	0.921 *	Mediator (Partial)
Mediation of mental health	0.52 *	0.29 × 0.35 = 0.101	0.621 *	Mediator (Full)
**Rural**				
Mediation of food intake	−0.29 *	−0.09 × 0.38 = 0.022	−0.312 *	-
Mediation of mental health	−0.29 *	0.25 × 0.41 = 0.102 *	−0.392 *	Mediator (Partial)

In this Table * is representative of significant relationship.

**Table 10:** Comparison analysis between rural and urban obesity modeling.

Path	Urban Model	Rural Model	Chin Test
Demographics → Lifestyle	0.39	0.11	2.36 *
Demographics → Food Intake	0.31	0.08	1.99 *
Demographics → Mental Health	0.47	0.12	3.31 *
Demographics → BMI	0.12	−0.14	2.07 *
Lifestyle → Food Intake	0.55	−0.02	7.21 *
Lifestyle → Mental Health	0.25	0.23	0.66
Lifestyle → BMI	0.56	−0.25	11.34 *
Food Intake → Mental Health	0.09	0.54	5.87 *
Food Intake → BMI	0.66	0.34	3.09 *
Mental Health → BMI	0.37	0.39	0.98

* Has significant difference with 95% confidence interval. Note: For moderation analysis, both groups (urban and rural) must have the same research variables. In this part of the analysis, we considered all of the research variables in both groups. Therefore, based on the factor loading analysis, we will not eliminate any indicators. In the above sections, we explained the differences among SEM with three types of estimators. There are two more comparison analyses between the SEM with correlation analysis and MANOVA, which we presented in the following two notes. *Note 1*. Correlation analysis is a common statistical method that some research scholars have been using it to understand the relationship between research variables. However, this method has some weaknesses. Correlation analysis only gives information about the relationship between two variables without involving other variables. Therefore, judging the relationship between two variables based on a correlation analysis would lead to a lack of accuracy. Based on Table 6, the normality of the research variables is rejected. Therefore, for correlation analysis, Spearman correlation was considered. Supplementary Materials Table S1 shows the outputs of the Spearman correlation between all the research variables. Supplementary Materials Table S1 shows the Spearman correlation between BMI and physical activity is equal to 0.019 (*p*-value = 0.684). It means there is no correlation between BMI and physical activity among our sample. However, according to Table 5, the factor loading of physical activity in lifestyle is equal to 0.77, and lifestyle has a significant correlation with BMI.

**Table 11:** Comparison analysis between SEM and MANOVA.

Index	Model 1	Model 2	Model 3	Model 4
R-Square of SEM	0.762	0.812	0.834	0.711
R-Square of MANOVA	0.699	0.702	0.821	0.684
RMSE of SEM	2.113	2.765	2.097	3.621
RMSE of MANOVA	2.222	3.431	4.127	3.881

## Supplementary Materials

The supplementary materials are available online at https://www.mdpi.com/1660-4601/17/14/5201/s1.
